# Multilocus Sequence Typing and Antimicrobial Resistance of *Campylobacter jejuni* Isolated from Dairy Calves in Austria

**DOI:** 10.3389/fmicb.2016.00072

**Published:** 2016-02-03

**Authors:** Daniela Klein-Jöbstl, Dmitri Sofka, Michael Iwersen, Marc Drillich, Friederike Hilbert

**Affiliations:** ^1^Clinical Unit for Herd Health Management, University Clinic for Ruminants, Department for Farm Animals and Veterinary Public Health, University of Veterinary Medicine ViennaVienna, Austria; ^2^Institute of Meat Hygiene, Meat Technology and Food Science, Department for Farm Animals and Veterinary Public Health, University of Veterinary Medicine ViennaVienna, Austria

**Keywords:** *Campylobacter jejuni*, dairy calf, MLST, antimicrobial resistance

## Abstract

Human campylobacteriosis is primarily associated with poultry but also cattle. In this study, 55 *Campylobacter jejuni* strains isolated from 382 dairy calves’ feces were differentiated by multilocus sequence typing and tested for antimicrobial resistance. The most prevalent sequence type (ST) was ST883 (20.0%), followed by ST48 (14.5%), and ST50 (9.1%). In contrast to ST48 and ST50, ST883 has rarely been described in cattle previously. Furthermore, risk factor analysis was performed for the presence of the most prevalent STs in these calves. Multiple regression analysis revealed that the type of farm (organic vs. conventional) and calf housing (place, and individual vs. group) were identified as significantly (*p* < 0.05) associated with the presence of ST883 in calves, and ST50 was associated with calf diarrhea. Antimicrobial resistance was detected in 58.2% of the isolates. Most of the resistant isolates (81.3%) were resistant to more than one antimicrobial. Most frequently, resistance to ciprofloxacin (49.1%), followed by nalidixic acid (42.8%), and tetracycline (14.5%) was observed. The results of the present study support the hypothesis that dairy calves may serve as a potential reservoir for *C. jejuni* and pose a risk for transmission, including antimicrobial resistant isolates to the environment and to humans.

## Introduction

*Campylobacter jejuni* is one of the most common causes of bacterial gastro-enteritis in humans and is of significant public health concern worldwide. Human campylobacteriosis is primarily associated with poultry, followed by cattle (French et al., 2009; de Haan et al., 2010b; Mughini Gras et al., 2012). Main risk factors are consumption of contaminated food, particularly poultry meat, raw milk, and water, as well as close contact to animals (Schildt et al., 2006; Heuvelink et al., 2009; Mughini Gras et al., 2012).

To distinguish between different *Campylobacter* strains, various methods have been applied, whereby multilocus sequence typing (MLST) has been identified as one of the best methods for application in epidemiological studies (Dingle et al., 2001; Korczak et al., 2009). The most commonly identified *C. jejuni* clonal complexes (CC) in bovines are CC21 and CC61. Sequence types (ST) of CC21 are not only typical for bovines, but also for other ruminants (sheep), poultry, and humans. In contrast, CC61 STs have been described as cattle associated (Kärenlampi et al., 2007; French et al., 2009; Grove-White et al., 2010; Bianchini et al., 2014), but are also frequently identified in humans, but not in poultry, suggesting that cattle may be an important source of human infection by contamination of food and water (French et al., 2009; Grove-White et al., 2010; Bianchini et al., 2014). Furthermore, CC42, CC45, CC48, and CC403 are frequently detected in cattle. Most of these CCs (CC42, CC45, and CC48) are also frequently identified in humans (French et al., 2009). These findings underline the importance of cattle in the epidemiology of human campylobacteriosis.

*Campylobacter* has been classified by the European Union as a zoonotic pathogen to be screened for antimicrobial resistance (Council directive, 2003/99/EC). However, this screening is limited to chicken and turkey isolates and does not include isolates from cattle. Key reasons for this are missing extensive European wide information on the risk of cattle isolates for human disease and low rates in antimicrobial resistance reported for cattle isolates (Aarestrup et al., 1997).

The aim of the present study was to evaluate different genotypes of *C. jejuni* in feces of preweaned calves in Austrian dairy herds by use of MLST and to investigate their antimicrobial resistance.

## Materials and Methods

### Samples

Fecal samples were collected from preweaned calves on 100 dairy farms in two Austrian regions (Lower Austria and Styria) during 2009 and 2010. This study was part of a study designed to examine differences between calves and farms with and without diarrhea (Klein et al., 2013; Klein-Jobstl et al., 2014). For selection of farms, local veterinarians were asked to provide lists with dairy farms with a documented problem of calf diarrhea during the last year. A farm with diarrhea problems was defined as a farm with multiple treatments for calf diarrhea by the veterinarian. Out of these lists, farms were randomly chosen. Additionally, farms from the same geographical region and of similar structure, with no history of calf diarrhea problems and no diarrheic calf at the time of sampling, were examined. In herds with five or less preweaned calves (which was the case on 62 of all farms), all calves were tested. In herds with more than five preweaned calves, five animals were randomly chosen. On the assumption of an inter-herd prevalence of over 40% (Ellis-Iversen et al., 2009) five samples were required from each herd to detect one positive calf with 95% confidence (calculation by use of Win Episcope 2.0^1^). Samples were taken directly from the rectum. Feces were placed in sterile plastic tubes and transported to the laboratory in coolers. Farm management characteristics were evaluated by a face to face interview by use of a questionnaire during the farm visit (**Table 1**; Klein et al., 2013). All sampled calves were examined clinically according to the clinical examination of ruminants (Radostits et al., 2007). Feces was evaluated as described by Larson et al. (1977), where score 3 and 4 were categorized as diarrheic. Furthermore, the calf rearing areas were inspected and hygiene was estimated by evaluation of calf housings (bedding and pen walls) and the calves themselves according to Lundborg et al. (2005).

**Table 1:** Variables surveyed on farm.

Area of interest	Variable
Farm characteristics	Region; production (organic vs. conventional); number of cattle and cows on farm; other farm animals than cattle on farm; if yes, which other farm animals, contact to other farm animals; workers on farm; other animals (companion animals) with access to the cows‘ and calves’ stable; water source
Housing	Housing of cows; pasture; calving area; calf housing (location, type, bedding)
Calf feeding	Colostrum management; milk feeding; feeding of hay, and concentrates; water
Hygiene	Cleaning and disinfection of different areas and barns; feed hygiene; cleaning of feeding equipment; milking hygiene
Miscellaneous	Dry off regime; dry period length
Individually sampled calves	Age (days); housing; feeding; diseases; treatments; treatment with antimicrobials; feeding of non-saleable milk

Results regarding risk factors for the presence of *C. jejuni* in calves were published elsewhere (Klein et al., 2013).

This study was discussed and approved by the institutional ethics committee of the University of Veterinary Medicine Vienna in accordance with Good Scientific Practice and national legislation.

### Laboratory Procedures

All fecal samples were processed within one day, held chilled until processing. Samples were prepared for detection and isolation of thermophilic *Campylobacter* according to standards described by ISO-10272-(2002). Briefly, after enrichment in Bolton Broth (Oxoid, Basingstoke, England) at 42°C for 48 h under microaerophilic conditions (10% CO_2_, 5% O_2_, and 85% N_2_), the samples were plated on two selective agars, modified CCDA (charcoal cefoperazon deoxycholate; Oxoid, Basingstoke, England) and CampyFoodAgar (Bio Merieux, Marcy l‘Etoile, France) and incubated at 42°C for 48 h under microaerophilic conditions. Additionally, all fecal samples were directly streaked onto the two selective agars without prior enrichment. One morphological typical colony per sample was differentiated by aerobic incubation, PCR (Linton et al., 1997) and 16S-rRNA-gene sequencing on selected strains.

### MLST

The MLST analysis was carried out as described by Dingle et al. (2001). Genomic DNA was extracted using a QIAamp DNA mini kit (Qiagen, Venlo, The Netherlands). The seven housekeeping loci defined by Dingle et al. (2001) as are *aspA, glnA, gltA, glyA, pgm, tkt*, and *uncA* were amplified using primers and protocols as described (Dingle et al., 2001). Sequencing was carried out by BigDye Terminator v3.1 cycle sequencing kit and an Applied Biosystems 310 ABI Prism genetic analyser. Sequence data were analyzed for MLST Types using the Campylobacter Multi Locus Sequence Typing website^2^ developed by Jolley and Maiden (2010) and funded by the Wellcome Trust.

### Antimicrobial Resistance Testing

Antimicrobial resistance was determined using CLSI M45-A^3^ for antimicrobial dilution and disk susceptibility testing of infrequently isolated or fastidious bacteria. Isolates were tested using disk susceptibility and the minimal inhibitory concentration was determined by antimicrobial dilution against ampicillin, amoxicillin/clavulanate, chloramphenicol, ciprofloxacin, colistin, erythromycin, gentamicin, nalidixic acid, neomycin, streptomycin, and tetracycline. As clinical breakpoints for *C. jejuni* are documented only for ciprofloxacin, erythromycin, and tetracycline (EUCAST) epidemiological cut-off-values, which have been determined by the European Committee on Antimicrobial Susceptibility Testing^4^, were applied on all *C. jejuni* isolates. For colistin and neomycin, no epidemiological cut-off-values have been determined. For these two antimicrobial substances cut-off were evaluated by comparing against values given in the literature and according to the distribution of our isolates were 16 mg/l for colistin and 4 mg/l for neomycin (El-Adawy et al., 2012; Ghimire et al., 2014).

### Detection of *gyrA* Mutations

In all quinolone resistant isolates the quinolone resistance-determining region (QRDR) of the *gyrA* gene a 220Bp PCR product was ampliefied with primers GyrA-for 5′-gctatgcaaaatgatgaggc-3′ and GyrA-rev 5′-cagtataacgcatcgcagcgg-3′ to detect the responsible point mutation at codon 86. Genomic DNA used for amplification was extracted as described above (MLST).

### Statistical Analysis

Data were statistically analyzed using PASW, version 20.0 (IBM Cooperation, New York, NY, USA).

The presence of each MLST ST was summarized in a binary variable. The presence of each ST was given as an individual variable, where presence of the given ST was categorized as one and not present as zero. Similarly, resistances against antimicrobials were categorized as either resistant (=1) or not resistant (=0). Different STs, resistances as well as farm characteristics or management factors were compared with the most prevalent STs and resistances against antimicrobials. Depending on the independent variable either Fisher’s exact test, Chi square test, binary logistic regression, or *t*-test was calculated. Correlation between *C. jejuni* MLST-types and resistance against antimicrobials were tested by Spearman correlation coefficient. The level of significance was set at a *p*-value of <0.05.

The presence of the most prevalent STs was associated with farm characteristics and management in a two-step process. First, the presence of the ST was compared to the different independent variables as described above by either Fisher’s exact test, Chi square test, binary logistic regression or *t*-test. All variables were tested for correlation among each other by Spearman correlation coefficient before entering the model. If a correlation between two variables >0.60 was given, one of the covariates was discarded taking biological plausibility into account. In a second step, variables with a *p*-value ≤0.20 were entered in a multiple logistic regression model. Confounding was monitored by the change in the coefficient of a variable after removing another variable (Dohoo et al., 2009). If the change of the estimates was ≥25% the removed variable was considered to have a potential confounding effect and was consequently forced into the model. Model fit was evaluated with the Hosmer–Lemeshow test for 10 groups.

## Results

In total, 382 calves were sampled on 100 farms. Mean herd size was 40 ± 29 dairy cows (varying between 5 and 223 cows). Mean herd size did not differ between *C. jejuni* positive and negative farms (*p* = 0.67).

The median age of the sampled animals was 17 days [25 and 75% interquartile range (IR) 10–28]. *C. jejuni* positive calves were as young as 3 days and up to 67 days (median 18, IR 11-36). From 382 fecal samples, 55 (14.4%) were positive for *C. jejuni*. Another four samples (1.0%) were positive for *C. coli*. On farm level, on 30 of the 100 farms at least one animal shed *C. jejuni*, whereas only on five of these 30 farms all sampled animals were positive.

### MLST of *C. jejuni* Isolates from Calves

The 55 *C. jejuni* isolates yielded 19 STs of which two were previously unreported. The isolates were assigned to eight clonal complexes (CC), dominated by three CCs (CC21, CC48, and CC206) that accounted for 74.5%. Half of the isolates (50.9%) belonged to CC21. The most prevalent STs were ST883 (20.0%), followed by ST48 (14.5%), and ST50 (9.1%; **Table 2**).

**Table 2:** *Campylobacter jejuni* MLST types among 55 isolates from preweand dairy calves.

CC	ST	N positive samples	N positive farms
21	21	4	4
	47	1	1
	50	5	4
	864	4	2
	883	11	6
	1943	3	1
22	22	1	1
	2497	3	2
42	42	2	1
	2580	1	1
45	45	1	1
48	48	8	6
206	122	2	1
	572	2	1
	6021	1	1
353	356	1	1
354	4899	3	2
	Unknown	2	2

On 24 of the 30 *C. jejuni* positive farms (80%) one ST was present. On five farms two and on one farm three different types were isolated.

### MLST Types and Risk Factors

The presence of *C. jejuni* ST883 in calves compared to the presence of other STs in calves was significantly associated with season, the presence of calf diarrhea on farm, the type of farm (organic vs. conventional), workers on farm, the feeding of waste milk, the separation of the calf from its dam after birth, calf feeding, and calf housing (individual versus group, and within cows’ barn versus outside the barn). These variables were entered in the multiple logistic regression model. As farm had a confounding effect, this variable was forced into the model. In the final model, the type of farm and calf housing pointed out to be significantly associated with the presence of ST883 in calves (**Table 3**). On conventional farms, the risk for calves to shed ST883 was lower compared with organic farms. Housing calves in groups inside the cows’ barn was identified as a risk for shedding ST883.

**Table 3:** Variables significantly associated with the presence of *C. jejuni* sequence type (ST) 883 in the final multiple logistic regression with farm forced into the model as a confounder.

		ST				
Variable		ST883	Others	OR^1^	95% CI^2^	*p*
Farm						0.45
Type of farm	Organic	6	6	1		
	Conventional	38	5	0.62	0.01-0.64	0.02
Calf housing	Individual	40	4	1		
	Group	4	7	23.16	2.08-257.43	0.01
	Within cows’ barn	15	9	1		
	Outside cows’ barn	29	2	0.78	0.01-0.93	0.04

*^1^OR = odds ratio*

*^2^CI = confidence interval*

*Hosmer–Lemeshow for the model *p* = 0.77.*

No significant associations were found with regard to the presence of ST48.

The presence of ST50 strains compared to other STs were associated with farm, type of farm (organic vs. conventional), farm size (number of cows on farm), the presence of poultry on farm, diarrhea, calf feeding, and antibiotic treatment in the calf. Farm was left in the multiple logistic model as it had a confounding effect. Finally, only one variable stayed significant in the final model. Calves suffering from diarrhea at the time of sampling had a higher risk to be ST50 positive than calves not suffering from diarrhea (OR 23.21, 95%CI 23.21-248.87, *p* = 0.01).

### Antimicrobial Resistance in *C. jejuni*

**FIGURE 1:**
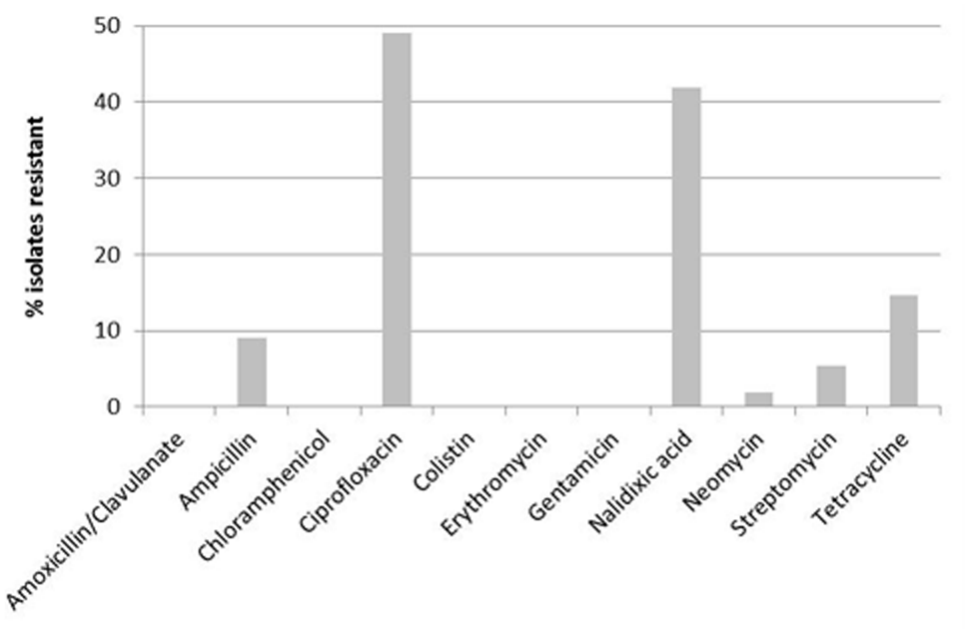
**Antimicrobial resistance of *Campylobacter jejuni* (*n* = 55) isolated from preweaned dairy calves**.

Of the 55 *C. jejuni* strains, 32 (58.2%) were resistant to at least one of the tested antimicrobials. Strains were resistant to ampicillin, ciprofloxacin, nalidixic acid, neomycin, streptomycin, and tetracycline (**Figure 1**). Twenty-six of the isolates (47.3%) were resistant to at least two of the tested antimicrobials. Seven of these isolates were resistant against three to five antimicrobials. Most frequently, resistance to ciprofloxacin was observed (49.1%), followed by nalidixic acid with 42.8%, and tetracycline (14.5%). No resistance was observed against amoxicillin/clavulanate, chloramphenicol, colistin, erythromycin, and gentamicin.

Associations could be observed in resistance against ciprofloxacin and nalidixic acid (*p* = 0.01), as well as against ciprofloxacin and ampicillin (*p* = 0.02). All isolates that were resistant against nalidixic acid or ampicillin were also resistant against ciprofloxacin.

### Genetic Identification of Quinolone Resistant Isolates

As almost half of the isolates were identified to be quinolone resistant in all of these isolates the QRDR of the *gyrA* gene was sequenced to detect the responsible point mutation at codon 86 the most important mechanism in *C. jejuni* for quinolone resistance. In all but one of the 27 isolates the point mutation at codon 86 (ACA to ATA) resulting in a substitution of isoleucine for threonine in gyrase A has been detected. One sensitive isolate sequenced confirmed the original sequence (ACA) as seen in sensitive isolates. Another mutation detected in three of the isolates at location codon 110 did not change the amino acid composition of the enzyme. One isolate resistant to ciprofloxacin and nalidixic acid did not have any mutation in the sequenced region.

### Antimicrobial Resistance and MLST-Types

Of the 11 STs that were detected repeatedly, in four STs (ST122, ST572, ST883, and ST4899) all isolates were ciprofloxacin resistant, whereas in ST883 this association was significant (*p* = 0.01). In ST42, ST864, ST1943, and ST2497, no resistance against ciprofloxacin were observed. Similar findings were obtained for nalidixic acid resistances, see **Table 4**.

**Table 4:** Antimicrobial resistance of the 55 *C. jejuni* MLST types obtained from dairy preweaned calves.

CC	ST	N isolates	N resistances	Antimicrobial resistance
21	21	2	2	CIP, NAL
	21	1	3	AMP, CIP, NAL
	21	1	1	STREP
	47	1	1	TET
	50	2	3	CIP, NAL, TET
	50	2	2	CIP, NAL
	50	1	0	
	864	2	1	TET
	864	2	0	
	883	9	2	CIP, NAL
	883	1	3	CIP, NAL, STREP
	883	1	3	AMP, CIP, NAL
	1943	3	0	
22	22	1	0	
	2497	3	0	
42	42	2	0	
	2580	1	0	
45	45	1	0	
48	48	7	0	
	48	1	2	CIP, NAL
206	122	2	2	CIP, NAL
	572	1	2	AMP, CIP
	572	1	3	AMP, CIP, NAL
	6021	1	5	AMP, CIP, NEO, STREP, TET
353	356	1	0	
354	4899	2	2	CIP, NAL
	4899	1	1	CIP
	Unknown	1	0	
	Unknown	1	1	TET

*CIP, ciprofloxacin; NAL, nalidixic acid; TET, tetracycline; AMP, ampicillin; STREP, streptomycin; NEO, neomycin.*

Testing for correlation between *C. jejuni* MLST-types and antimicrobial resistance revealed significant associations between ST48 and ciprofloxacin resistance (coefficient = –0.33; *p* = 0.01), between ST572 and resistance against ampicillin (coefficient = –0.61; *p* < 0.01), between ST864 and ciprofloxacin (coefficient = –0.28; *p* = 0.04) and tetracycline (coefficient = 0.28; *p* = 0.04) resistance, and between ST883 and ciprofloxacin and nalidixic acid, respectively (coefficient = 0.51; *p* < 0.01).

Only two of the 55 *C. jejuni* positive calves were previously treated with antimicrobials. Both of these calves shed antimicrobial resistant *C. jejuni* (ST50). Both strains were resistant to ciprofloxacin and nalidixic acid, one additionally against tetracycline.

There was no association between the evaluated use of antimicrobials on farm and resistances.

## Discussion

To the knowledge of the authors studies examining *C. jejuni* MLST types in cattle have been limited to quinolone-resistant *C. jejuni* in Austria (Kovac et al., 2015) and studies in dairy calves are sparse.

*Campylobacter jejuni* isolates detected in the calves of our study were dominated by CC21, a CC regularly associated with cattle (Manning et al., 2003; Kwan et al., 2008; Ragimbeau et al., 2008; de Haan et al., 2010b; Bianchini et al., 2014; Jonas et al., 2015). Ten of the 19 *C. jejuni* STs detected were previously described in cattle. ST 883, the most common ST in our study, has only been described in cattle sporadically (de Haan et al., 2010b; Bianchini et al., 2014), and was furthermore sporadically associated with poultry and human campylobacteriosis (Wirz et al., 2010; Kittl et al., 2013b). In the study by Kovac et al. (2015) where ciprofloxacin-resistant *C. jejuni* of 17 cattle from Austria were examined, only one was ST883. In contrast, ST48 and ST50, the second and third most prevalent STs in the present study, were commonly associated with bovines, including Austria (Kwan et al., 2008; Ragimbeau et al., 2008; Rapp et al., 2014; Kovac et al., 2015). This ST was also associated with humans and other species, e.g., poultry (Ragimbeau et al., 2008; de Haan et al., 2010a). In the PubMLST database so far 32 human isolates and 33 chicken isolates from Austria have been downloaded between 2008 and 2014. Interestingly CC21 the CC most prevalent in claves in our study was also dominant in human isolates in Austria (21% of all isolates) whereas the clonal complex CC464, CC353, and CC354 dominated in chicken isolates.

Different studies emphasized the role of cattle in human campylobacteriosis. Following poultry, bovines were frequently associated with human infections (Wilson et al., 2009; de Haan et al., 2010a; Mughini Gras et al., 2012). Risk factors that have been described are direct contact to cattle and to cattle feces, as well as consumption of raw milk (Eberhart-Phillips et al., 1997; Smith et al., 2004; Schildt et al., 2006). Direct contact and consumption of raw milk is given not only for farmers but also, e.g., during farm vacation which is popular in Austria with nearly 10,000 farms offering this service (Grüner Bericht, n.d.). Furthermore, contaminated food or water can play a role in human infection (Clark et al., 2005; Levesque et al., 2008; Mughini Gras et al., 2012). The three dominating CCs (CC21, CC48, and CC206) and STs (ST883, ST48, and ST50) of our study were also recovered from infected humans (Ragimbeau et al., 2008; Mullner et al., 2009; Sheppard et al., 2009; de Haan et al., 2010a). This result indicates that calves may be a potential source of human infection, but this cannot be proven by this study. Gilpin et al. (2008) found indistinguishable *C. jejuni* genotypes in dairy calves and humans, using Penner serotyping and pulsed field gel electrophoreses, and came to the same conclusion, that calves may be a source of human campylobacteriosis.

Early campylobacter infection in calves might be due to a high level of environmental contamination, as well as direct contact with feces and ingestion of milk (Bianchini et al., 2014). Contamination of calf housings represents a risk for early infection with *C. jejuni* ST883. Calves housed within the cows‘ barn were at higher risk to shed ST883 than calves housed in a special barn for calves and young animals or outside the barn. Additionally, grouping of animals was a risk factor for shedding ST883. These findings suggest that close contact to adult as well as to other young cattle lead to higher infection pressure and mutual infection between animals. Furthermore, grouping can be a stress factor and consequently lead to a higher rate of campylobacter shedding and infection (Rapp et al., 2014).

Furthermore, ST883 was associated with the type of farm. On conventional farms, the risk for preweaned calves to be ST883 positive was lower than on organic farms, a finding that can hardly be explained. Possibly a certain clone is circulating on these farms and has been established.

The presence of ST50 was associated with diarrhea at the time of sampling in calves. Although some authors (Al-Mashat and Taylor, 1983; Diker et al., 1990; Schulze, 1992) suggested a possible role of *Campylobacter* in calf diarrhea, in other studies no association between *C jejuni* and disease was given (De Rycke et al., 1986; Acha et al., 2004), as was also true for the calves of the present study (Klein et al., 2013) and more likely other pathogens generally associated with calf diarrhea may be the cause of disease.

More than half of the calves (55%) originated from farms where also poultry was kept. Most of the STs detected in the calves of our study were also described in poultry. In the present study, specific types like ST50, frequently detected in poultry (de Haan et al., 2010b; Griekspoor et al., 2010; Kovanen et al., 2014), were associated with the presence of poultry on farm, suggesting cross-contamination between the two species. In contrast, other STs (e.g., ST21, ST48, and ST883) also regularly detected in poultry (Wirz et al., 2010; Kittl et al., 2013b) appeared in calves independently of the presence of poultry.

Antimicrobial resistance, particularly multidrug resistance is of public health concern. In the present study, 58.2% of the *C. jejuni* isolates were resistant to at least one and 47.3% against at least two of the tested antimicrobials. Because only two of the 55 *C. jejuni* positive calves were previously treated with antibiotics, no valid conclusion can be drawn if resistance to STs in this study was associated with previous antibiotic treatment. Nevertheless, these two treated animals shed *C. jejuni* resistant to two and three of the tested antimicrobials, respectively. In the present study, antimicrobial resistance to quinolones was detected most often. This has also been described for isolates originating from other sources (Oporto et al., 2009; Kittl et al., 2013a), explained by the fact that a single mutation is sufficient to cause resistance (Wang et al., 1993). This was confirmed in our study as all but one resistant isolates harbored a point mutation in codon 86 of the *gyrA* gene. Quinolone resistance has been described to be associated with specific ST types as detected in our study (Kittl et al., 2013a; Kovac et al., 2015). A survey performed in Austria revealed that quinolones are frequently used by Austrian veterinarians for treatment of cattle (Mayrhofer et al., 2006). This might explain a high level of quinolone resistance in *C. jejuni* isolates from calves.

## Conclusion

The results of the present study support the hypothesis that cattle including dairy calves may be a reservoir for *C. jejuni* and represent a risk for transmission of these bacteria to the environment and to humans. Cattle have not been recognized as an important source for antimicrobial resistant *Campylobacter* sp. or other bacteria, yet. Nevertheless, high resistance rates found in this and other recent studies point out that screening for antimicrobial resistance in cattle is necessary to better understand the epidemiology of resistance and its spread.

## Author Contributions

DK-J designed the study, took all samples, performed statistical analysis, and drafted the manuscript. DS performed laboratory work and analysis. MI advised statistical analysis and interpretation, and reviewed the manuscript. MD supported the statistical analysis and reviewed the manuscript. FH designed the study together with DK-J and supervised the study, supervised, and performed laboratory work and analysis, and provided valuable references and suggestions during the preparation of the manuscript.

## Conflict of Interest Statement

The authors declare that the research was conducted in the absence of any commercial or financial relationships that could be construed as a potential conflict of interest.

## Footnotes

1 http://www.winepi.net/uk/index.html

2 http://pubmlst.org/campylobacter/

3 www.clsi.org

4 www.eucast.org
